# Prevalence and Determinants of the Co-Occurrence of Overweight or Obesity and Micronutrient Deficiencies among Adults in the Philippines: Results from a National Representative Survey

**DOI:** 10.3390/nu13072339

**Published:** 2021-07-08

**Authors:** Aileen R. de Juras, Wan-Chen Hsu, Susan C. Hu

**Affiliations:** 1 Department of Public Health, College of Medicine, National Cheng Kung University, Tainan City 701, Taiwan; ardejuras@up.edu.ph (A.R.d.J.); jenny13929@gmail.com (W.-C.H.); 2 Institute of Human Nutrition and Food, College of Human Ecology, University of the Philippines Los Baños, Laguna 4031, Philippines

**Keywords:** double burden of malnutrition, overweight, obesity, micronutrient deficiency, adults, the Philippines

## Abstract

The double burden of malnutrition (DBM) affects many low- and middle-income countries. However, few studies have examined DBM at the individual level, or undernutrition and overnutrition co-occurring within the same person. This study aims to investigate the prevalence and determinants of DBM among adults in the Philippines. Data from the 2013 National Nutrition Survey were used. The sample size in the analysis was 17,010 adults aged ≥20 years old, after excluding pregnant and lactating women. DBM was defined as the co-occurrence of overweight/obesity and anemia (definition #1), overweight/obesity and anemia or vitamin A deficiency (definition #2), and overweight/obesity and anemia or vitamin A deficiency or iodine insufficiency (definition #3). Anthropometric measurements and biochemical markers were used for DBM assessment. Data were analyzed using descriptive statistics, the chi-square test, and logistic regression by R software. Results showed that definition #3 is the predominant type of DBM (7.0%) in the general population, whereas the prevalence of DBM has increased to 23.7% in overweight/obese persons. Sex, age, educational attainment, marital status, household size, wealth quintile, and smoking status were the determinants of DBM. This study revealed that Filipino adults experience malnutrition critically and must be addressed through food and nutrition interventions.

## 1. Introduction

The double burden of malnutrition (DBM) is a public health challenge confronting low- and middle-income countries [1]. According to the definition from the World Health Organization (WHO), DBM is a phenomenon of “the coexistence of undernutrition or micronutrient deficiency along with overweight, obesity or diet-related non-communicable diseases” [2]. DBM can take place at three levels. The first level is individual, wherein there is the simultaneous development of two or more types of malnutrition within the same person, for example, obesity with anemia or any vitamin or mineral deficiencies or insufficiencies. Second, at the household level, DBM manifests when malnutrition co-exists among parents and their children. Finally, the population is the third level, and DBM occurs when undernutrition and overnutrition or diet-related non-communicable diseases (NCDs) are observed in the community, region, or country [2].

DBM, also called “malnutrition in all its forms,” may be temporally separated. It can happen across the life course; for example, an obese adult may be stunted during their childhood [2] and thus has grave health and economic consequences [3,4]. Therefore, ending DBM is a global goal under the United Nations Decade of Action of Nutrition, and one key action to address this problem is an understanding of its prevalence and distribution at all levels [2,5].

Most past studies investigating DBM have focused on the national and household levels. Limited research has been conducted at the individual level. Moreover, the published literature on individual-level DBM among adults has mainly examined the co-occurrence of overweight/obesity and anemia or vitamin A deficiency [6,7,8,9,10,11]. Characterizing DBM using anemia and vitamin A deficiency as the only measures of micronutrient deficiency may be constrained because, in other countries, iodine deficiency is also of public health significance. Thus, further examination of other micronutrient deficiencies overlapping with overweight/obesity is vital to explain the full scale of the DBM problem.

The Philippines, a lower-middle-income country in Asia, is facing the double burden of malnutrition. This was shown in the 8th National Nutrition Survey results, wherein the prevalence of overweight/obesity and micronutrient deficiencies (such as vitamin A deficiency, anemia, and iodine deficiency) was high among adults at the national level [12]. However, DBM at the individual level or the co-occurrence of overweight/obesity with micronutrient deficiencies within the same person has received little attention in the Philippines. Therefore, the present study aims to (1) describe the prevalence of three types of DBM at the individual level among Filipino adults (#1: the co-occurrence of overweight/obesity and anemia; #2: the co-occurrence of overweight/obesity and anemia or vitamin A deficiency; and #3: the co-occurrence of overweight/obesity and anemia or vitamin A deficiency or iodine insufficiency) and (2) examine the association between DBM and sociodemographic and health-related factors.

## 2. Materials and Methods

### 2.1. Data Source and Study Population

This study used data from the latest 2013 Philippine National Nutrition Survey (PNNS), a cross-sectional and population-based survey carried out by the Department of Science and Technology–Food and Nutrition Research Institute (DOST-FNRI). The 2013 PNNS adopted the 2003 Master Sample of the National Statistics Office and employed a three-stage stratified sampling design covering 17 regions and 80 provinces of the country [13]. Details of the survey history, objectives, design, and methods are reported elsewhere [14].

**Figure 1:**
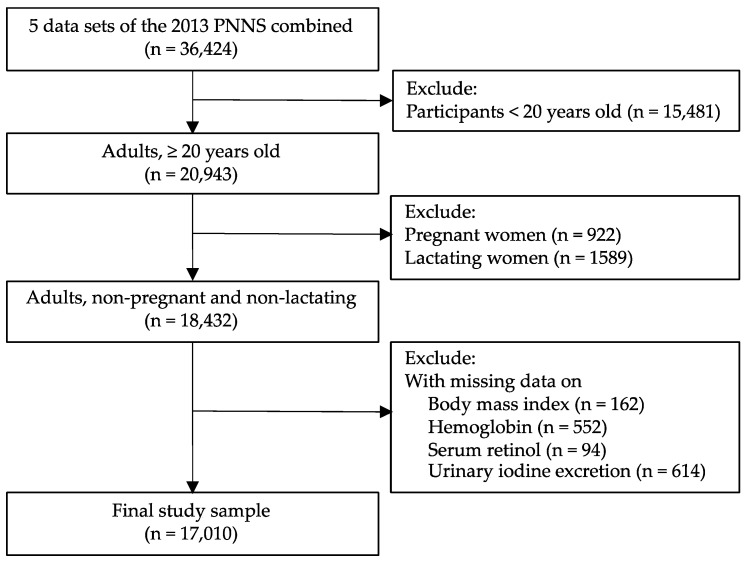
Flowchart of participant selection.

Data from five survey components were used in the analysis: (1) anthropometry, (2) biochemical, (3) clinical, (4) socio-economic individual-level, and (5) socio-economic household-level components; these are available online at the DOST-FNRI eNutrition website (http://enutrition.fnri.dost.gov.ph/site/home.php (accessed on 3 September 2020)). Dietary data were available in the 2013 PNNS but were beyond the scope of the current study. From the 36,424 eligible participants with complete subject identification data in the five components, those less than 20 years old (*n* = 15,481), pregnant (*n* = 922), and lactating (*n* = 1589) were excluded. Study participants with missing values on the body mass index (*n* = 162), hemoglobin (*n* = 552), serum retinol (*n* = 94), and urinary iodine excretion (*n* = 614) were also excluded from the analysis. The final study sample consisted of 17,010 adults (8536 men and 8474 non-pregnant and non-lactating women) (Figure 1).

### 2.2. Sociodemographic Characteristics and Health-Related Behavior Measurement

Sex, age, educational attainment, marital status, employment status, household size, and wealth quintile were the sociodemographic factors assessed in this study. The sex of the participants was classified as male or female. The age variable was grouped as 20–39, 40–59, and ≥60 years old. Educational attainment was categorized as elementary and lower, high school, and college and higher. Marital status was divided into three categories: single, married or with a partner, and others (widowed/separated/annulled/divorced). Employment status was reported as a binary variable (employed or unemployed). Household size was created from the socio-economic datasets and classified into three levels. Wealth status was determined from the household assets, household characteristics, access to utilities, and infrastructure variables through principal component analysis. The resulting scores in the analysis were equally divided into five groups to derive the wealth quintiles (poorest, poor, middle, rich, and richest) [12].

Smoking, alcohol consumption, and physical activity constituted health-related behaviors. The categories for smoking status and physical activity were according to the WHO STEPwise Approach to Surveillance manual [15]. Current smokers were denoted by smoking either (a) daily, at least one cigarette a day or on a regular/occasional basis, or (b) not daily, but at least weekly or less often than weekly during the survey. Low physical activity was indicated as not engaging in either (a) 3 or more days of vigorous-intensity activity for at least 20 min per day or (b) 5 or more days of moderate-intensity activity or walking for at least 30 min per day. Current alcohol drinkers, measured based on the WHO guidelines, pertained to the consumption of any alcoholic beverage in the past 12 months [16]. The above-mentioned sociodemographic characteristics and health-related behaviors were collected via face-to-face interviews by trained survey researchers [12].

### 2.3. Anthropometric and Biochemical Assessments

Weight and height were the anthropometric measurements included in the present study. The weight was measured using the Detecto™ platform beam balance weighing scale and recorded to the nearest 0.1 kg. The height was obtained using the Seca™ microtoise and documented to the nearest 0.1 cm. Weight and height measurements were taken twice in the survey [12]. The weight in kilograms was divided by the height in meters squared to calculate the body mass index (BMI). BMI categories were interpreted based on the WHO reference standard as underweight (<18.5 kg/m^2^), normal weight (18.5–24.9 kg/m^2^), overweight (25.0–29.9 kg/m^2^), and obese (≥30.0 kg/m^2^) [17].

The PNNS provides biochemical data for anemia, vitamin A deficiency, and iodine insufficiency determination. Venous blood samples were collected from the study participants by licensed medical technologists to assess anemia and vitamin A deficiency. From the blood samples, hemoglobin was determined employing the cyanmethemoglobin method of the International Committee for Standardization in Hematology (1978) [18], and anemia was defined as hemoglobin < 13 g/dL for males and <12 g/dL for females [19]. Vitamin A deficiency was evaluated using serum retinol extracted from the blood collected. The high-performance liquid chromatography method of Furr et al. (1992) [20] was used to examine serum retinol with a cut-off of <10 μg/dL for vitamin A deficiency [21]. Urine samples were analyzed for urinary iodine excretion (UIE) concentrations through the acid digestion/colorimetric method of Dunn et al. (1993) [22]. A UIE of <50 μg/dL indicated iodine insufficiency [23]. All biochemical analyses were carried out at the Biochemical Laboratory of the Food and Nutrition Research Institute, Department of Science Technology, the Philippines [12].

### 2.4. Double Burden of Malnutrition Definitions

Three definitions were used to characterize DBM at the individual level among Filipino adults: (#1) the co-occurrence of overweight/obesity and anemia (Ow/Ob + Ane), (#2) the co-occurrence of overweight/obesity and anemia or vitamin A deficiency (Ow/Ob + Ane/VAD), and (#3) the co-occurrence of overweight/obesity and anemia or vitamin A deficiency or iodine insufficiency (Ow/Ob + Ane/VAD/Iod-Ins). These definitions are also referred to as DBM types in this paper.

### 2.5. Statistical Analysis

Descriptive statistics were generated for the sociodemographic characteristics and health-related behaviors of adults. The prevalence of overweight, obesity, anemia, vitamin A deficiency, iodine insufficiency, and DBM definitions was calculated. Differences between sex and other independent variables were assessed using the chi-square test. A similar analysis was carried out to show variations in DBM types according to sociodemographic and health-related variables. Only definitions #1 and #3 were included in the analysis because the prevalence of Ow/Ob + Ane/VAD (definition #2) was the same as that of Ow/Ob + Ane (definition #1).

The association of sociodemographic characteristics and health-related behaviors with each type of DBM was examined using binary logistic regression. In addition, multicollinearity among the independent variables was tested by evaluating variance inflation factors. All analyses were conducted using R version 4.0.3 and employed survey weights to generate results representative of adults in the Philippines. Survey procedures in R were used to account for complex sampling design. The level of significance was set at *p* < 0.05.

## 3. Results

### 3.1. Participants’ Characteristics

The characteristics of the study participants stratified by sex are presented in Table 1. Males composed 50.1% of the study sample and were mostly (48.8%) in the 20–39-year age group. In contrast, females composed 49.9% of the participants, and 43.2% were aged between 20 and 39 years. The level of education was low (70.2% with high school education and below). Slightly more females (31.9%) attained college education or higher than males (27.8%). Most participants were married or with sexual partners (68.3% for males and 65.8% for females). Employment status was statistically different between males and females, with more females (56.8%) being unemployed. The median household size was four, and no sex difference was observed. About 22% of the households belonged to the richest wealth quintile. There were more males than females in the poorest quintile and the opposite in the richest quintile. In terms of health-related behaviors, 27.2% were current smokers, 51.6% were current alcohol drinkers, and 43.3% had low physical activity. More males were smokers and drinkers, whereas more females had low physical activity.

Table 2 displays the nutritional status of Filipino adults by sex. Using the WHO BMI classification, 59.3% had normal weight, while 11.2% were underweight. Approximately one in five adults was overweight (23.4%), and 6.2% were obese. The prevalence of micronutrient deficiency/insufficiency varied. Iodine insufficiency had the highest prevalence (23.8%), followed by anemia (6.5%) and then vitamin A deficiency (0.1%). There were sex differences in the adults’ nutritional status, with females having a higher prevalence of underweight, overweight, obesity, anemia, and iodine insufficiency.

### 3.2. Prevalence of the Double Burden of Malnutrition

The prevalence of the three types of DBM in the general population was 1.4% for definition #1 (co-occurrence of overweight/obesity and anemia), 1.4% for definition #2 (co-occurrence of overweight/obesity and anemia or vitamin A deficiency), and 7.0% for definition #3 (co-occurrence of overweight/obesity and anemia or vitamin A deficiency or iodine insufficiency). Definitions #1 and #2 had the same prevalence, and the third definition was the most common type of DBM among Filipino adults (Table 2). Noticeably, females had a much higher prevalence than males in all forms.

**Figure 2:**
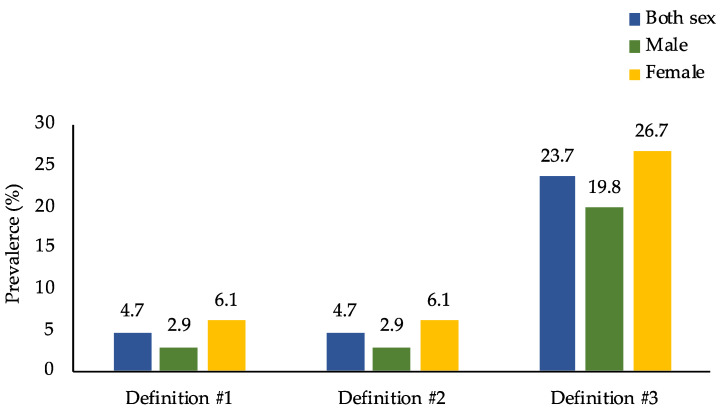
Prevalence of the co-occurrence of overweight/obesity and micronutrient deficiencies among overweight/obese adults in the Philippines by sex (*n* = 4816). Prevalence estimates accounted for the complex sampling design. Definition #1: overweight/obesity and anemia. Definition #2: overweight/obesity and anemia or vitamin A deficiency. Definition #3: overweight/obesity and anemia or vitamin A deficiency or iodine insufficiency.

**Figure 3:**
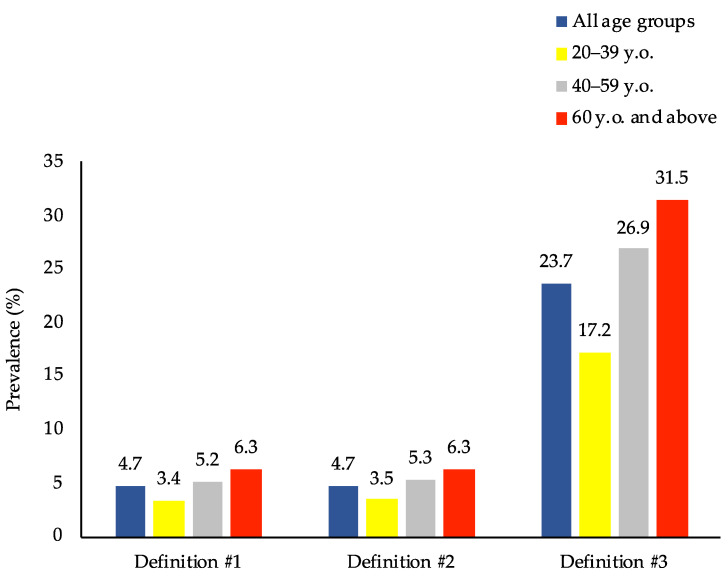
Prevalence of the co-occurrence of overweight/obesity and micronutrient deficiencies among overweight/obese adults in the Philippines by age group (*n* = 4816). Prevalence estimates accounted for the complex sampling design. Definition #1: overweight/obesity and anemia. Definition #2: overweight/obesity and anemia or vitamin A deficiency. Definition #3: overweight/obesity and anemia or vitamin A deficiency or iodine insufficiency.

However, when looking at overweight/obese persons only (*n* = 4816), Figure 2 and Figure 3 show that the prevalence of DBM was much higher than that in the general population, where the prevalence of DBM was 4.7%, 4.7%, and 23.7% for definitions #1, #2, and #3, respectively. Moreover, when disaggregated into sex and age groups, females and adults ≥ 60 years old were found to have the highest DBM prevalence across the three definitions.

### 3.3. Factors Associated with the Double Burden of Malnutrition

The distribution of DBM definitions #1 and #3 according to sociodemographic and health-related factors is summarized in Table 3. The prevalence of the two types of DBM was higher in females, 40–59 years old, and widowed/separated/annulled/divorced. In addition, participants living in households in the rich and richest wealth quintiles and those not currently smoking and drinking alcohol had a significantly higher prevalence for both DBM types. Unemployed adults were identified as related to a higher prevalence of Ow/Ob + Ane (definition #1).

### 3.4. Determinants of the Double Burden of Malnutrition

Several factors were associated with the two definitions of DBM (Table 4). The odds of Ow/Ob + Ane (definition #1) and Ow/Ob + Ane/VAD/Iod-Ins (definition #3) were significantly higher for females than for males. Middle-aged adults (40–59 years) had an increased risk than younger adults (20–39 years). Furthermore, those who were married or widowed/separated/annulled/divorced were twice likely to have DBM compared with single or unmarried adults. Finally, wealth status was associated with the two types of DBM. Surprisingly, adults dwelling in households in the rich and richest wealth quintiles had increased odds of experiencing these outcomes than those in the poorest wealth quintile, with a significant dose–response relationship.

Being in the older age group (60 years and above), having a high school education, belonging to households in the poor and middle-wealth quintiles, and not currently smoking were additional variables associated with Ow/Ob + Ane/VAD/Iod-Ins (definition #3). Noticeably, the middle household size was a protective factor in DBM definition #3, indicating that adults who reside in households with 4–6 members have lower risks of having these conditions.

## 4. Discussion

### 4.1. Main Findings

This study demonstrated that the adult population in the Philippines is facing malnutrition in all its forms. First, the prevalence of overweight/obesity was 29.6%, and that of anemia and iodine insufficiency was 6.5% and 23.8%, respectively. A tiny percentage of adults (0.1%) had vitamin A deficiency. Second, the most common type of DBM was the co-occurrence of overweight/obesity and anemia or vitamin A deficiency or iodine insufficiency, with 7.0% of Filipino adults suffering from it. However, when focusing on overweight/obese people only, the prevalence increased dramatically to more than three times (23.7%), wherein females had a much higher prevalence than males. Finally, the determinants of DBM were sex, age, educational attainment, marital status, household size, wealth quintile, and smoking status.

### 4.2. Interpretations

The three DBM types were observed among Filipino adults, with Ow/Ob + Ane/VAD/Iod-Ins (definition #3) as the most common. This was mainly due to the high prevalence of iodine insufficiency than anemia and vitamin A deficiency. Comparing prevalence estimates with other countries is quite complex due to limited published research and varying definitions used to measure this phenomenon. Nonetheless, Little et al. found that 18.6% of Indian adults simultaneously experience overweight/obesity and anemia [8]. In Burkina Faso, the dual burden of overweight/obesity and at least one deficiency in either iron or vitamin A among adults was reported at 8.5% [11]. Another study among Tajik women, but not men, evaluated the concurrence of overweight/obesity and micronutrient deficiencies, including anemia, vitamin A deficiency, and iodine deficiency; however, no significant DBM was seen among the women [24]. The prevalence of Ow/Ob + Ane (definition #1) and Ow/Ob + Ane/VAD (definition #2) among Filipino adults was lower than the findings mentioned above ascribable to the higher levels of Ow/Ob, anemia, and vitamin A deficiency in these countries. Moreover, DBM estimates may be influenced by the stage of nutrition transition a country is undergoing. Burkina Faso is in the early transition phase, whereas the Philippines has shifted toward a nutrition-related non-communicable disease pattern [25,26].

The co-occurrence of overweight/obesity and anemia, vitamin A deficiency, or iodine insufficiency affects adults disproportionately. Being a woman and older would increase the odds of both Ow/Ob + Ane and Ow/Ob + Ane/VAD/Iod-Ins. Filipino women are at a higher independent risk for overweight/obesity, anemia, vitamin A deficiency, and iodine insufficiency [12]. Adults’ age as a DBM-determining factor is due to the biological changes of increased fat mass and decreased basal metabolic rate that contribute to obesity development [27]. Similarly, food intake deteriorates as individuals become older, leading to excess or deficiency of energy and nutrients [12]. It should be noted that adults aged ≥ 60 years had a lesser risk for DBM than those in the 40–59-year age group. This finding may be a result of the lower overweight/obesity prevalence among older adults.

The socio-economic indicators, such as educational attainment, household size, and wealth quintile, were also correlated with the co-occurrence of overweight/obesity and micronutrient deficiencies. A possible explanation is a link between socio-economic status and food intake and diet diversity. Education can influence an individual’s dietary intake through having access to food and health information. Likewise, food intake can be altered by household size. According to the 2013 PNNS, the amount of food consumed is highest among households with fewer members and declines with increasing household size [12]. Wealth status was related to diets low in nutrients among Filipino adults [28]. To add, one of the cost-effective programs in addressing iodine deficiency is salt iodization. Although 71.5% of Philippine households were aware of iodized salt, less than half (47.5%) claimed to use it. Non-usage of iodized salt was due to taste difference, unavailability, and higher cost than ordinary salt. Furthermore, in the dietary survey component, non-iodized salt ranked as one of the topmost commonly consumed food items across all wealth quintiles [12]. The mechanisms through which marital status influences DBM is anchored on the availability of resources, the perception of attractiveness, and the presence of stresses [29].

Regarding the relationship between health-related behaviors and DBM, only non-smokers were observed to have higher odds of Ow/Ob + Ane/VAD/Iod-Ins than current smokers. Adults who are current smokers differ in the BMI from those who have never smoked. Smoking limits weight gain by decreasing food intake and increasing energy expenditure [30]. Collectively, these findings were consistent with the previous literature. Evidence from India and Tunisia corroborates that Ow/Ob + Ane affects a large proportion of women, married adults, individuals residing in households in a higher wealth quintile, and non-current smokers [8,9]. Past research examining overweight/obesity and micronutrient deficiencies using national surveys revealed that older age, higher education, and better socio-economic status are significant correlates [31]. Albeit this, caution in the interpretation of findings is necessary, given that the latter study covered only women of reproductive age.

### 4.3. Strengths and Limitations of the Study

A major strength of this study was its evaluation of DBM using a nationally representative dataset from the Philippines. In addition, biochemical markers for specific micronutrients collected in the 2013 PNNS were used to detect deficiency/insufficiency. In addition, three definitions of the co-occurrence of overnutrition (overweight/obesity) and micronutrient deficiency/insufficiency (including anemia, vitamin A deficiency, and iodine insufficiency) were examined to broaden the DBM characterization. However, the present study also had some limitations. First, its cross-sectional nature did not allow for life course analysis of DBM and for any causal inference to be drawn. Second, recall bias may have existed from self-reported health-related behaviors. Third, it is possible that unmeasured non-nutritional determinants, such as inflammation, may have contributed to DBM development. Finally, the missing data for the BMI, hemoglobin, serum retinol, and urinary iodine excretion constituted 7.7% of the overall sample; their exclusion unlikely, though possibly, biased the findings.

## 5. Conclusions

The double burden of malnutrition exists among Filipino adults. Overweight/obesity co-occurring with anemia or vitamin A deficiency or iodine insufficiency has the highest prevalence among the DBM types. Sex, age, educational attainment, marital status, household size, wealth quintile, and smoking status are the key determinants of DBM. These risk factors affect the simultaneous existence of overnutrition and micronutrient deficiency/insufficiency through unhealthy dietary and lifestyle practices. This study provides evidence suggesting that double-duty interventions are needed to address malnutrition in all its forms among adults in the Philippines.

## Figures and Tables

**Table 1:** Characteristics of study participants ^1^.

Characteristics	Total	Male	Female	*p*-Value
	(*n* = 17,010)	(*n* = 8536)	(*n* = 8474)	
Sex				-
Male	50.1	-	-	
Female	49.9	-	-	
Age group (years)				<0.001
20–39	46.0	48.8	43.2	
40–59	38.8	37.4	40.1	
≥60	15.2	13.8	16.7	
Educational attainment ^2^				0.001
Elementary and lower	31.9	33.7	30.0	
High school	38.3	38.5	38.1	
College and higher	29.8	27.8	31.9	
Marital status				<0.001
Single	23.3	26.5	20.1	
Married	67.1	68.3	65.8	
Others	9.6	5.2	14.0	
Employment status				<0.001
Employed	60.2	77.2	43.2	
Unemployed	39.8	22.8	56.8	
Household size				0.309
1–3	33.1	32.9	33.4	
4–6	44.5	44.4	44.7	
≥7	22.3	22.8	21.9	
Wealth quintile				<0.001
Poorest	17.3	19.1	15.6	
Poor	19.3	20.3	18.3	
Middle	20.6	20.7	20.4	
Rich	21.2	20.3	22.1	
Richest	21.6	19.6	23.6	
Current smoker ^2^				<0.001
Yes	27.2	46.2	8.2	
No	72.8	53.8	91.8	
Current alcohol drinker ^2^				<0.001
Yes	51.6	72.7	30.5	
No	48.4	27.3	69.5	
Physical activity ^2^				<0.001
Low	43.3	35.4	51.2	
High	56.7	64.6	48.8	

^1^ Data are weighted percentage (%). ^2^ Number of adults with missing data by variable: educational attainment (*n* = 71), smoking and alcohol drinking status (*n* = 974), and physical activity classification (*n* = 1163).

**Table 2:** Nutritional status of adults in the Philippines ^1^.

Variables	Total	Male	Female	*p*-Value
	(*n* = 17,010)	(*n* = 8536)	(*n* = 8474)	
Body mass index				<0.001
Underweight	11.2	10.6	11.8	
Normal weight	59.3	63.2	55.3	
Overweight	23.4	21.5	25.3	
Obese	6.2	4.6	7.7	
Anemia				0.002
Yes	6.5	5.2	7.7	
No	93.5	94.8	92.3	
Vitamin A deficiency				0.527
Yes	0.1	0.1	0.1	
No	99.9	99.9	99.9	
Iodine insufficiency				0.001
Yes	23.8	21.5	26.2	
No	76.2	78.5	73.8	
Co-occurrence of Ow/Ob and micronutrient deficiencies				
#1. Ow/Ob + Ane ^2^				0.001
Yes	1.4	0.8	2.0	
No	98.6	99.2	98.0	
#2. Ow/Ob + Ane/VAD ^3^				0.001
Yes	1.4	0.8	2.0	
No	98.6	99.2	98.0	
#3. Ow/Ob + Ane/VAD/Iod-Ins ^4^				<0.001
Yes	7.0	5.2	8.8	
No	93.0	94.8	91.2	

^1^ Data are weighted percentage (%). ^2^ Ow/Ob + Ane: overweight/obesity and anemia. ^3^ Ow/Ob + Ane/VAD: overweight/obesity and anemia or vitamin A deficiency. ^4^ Ow/Ob + Ane/VAD/Iod-Ins: overweight/obesity and anemia or vitamin A deficiency or iodine insufficiency.

**Table 3:** Bivariate analysis for factors related to DBM in adults in the Philippines ^1^.

Characteristics	Definition #1 Ow/Ob + Ane ^2^		Definition #3 Ow/Ob + Ane/VAD/Iod-Ins ^3^	
	(*n* = 237)	*p*-Value	(*n* = 1241)	*p*-Value
Sex		0.001		<0.001
Male	0.8		5.2	
Female	2.0		8.8	
Age group (years)		0.002		<0.001
20–39	0.9		4.4	
40–59	1.9		9.8	
≥60	1.6		7.9	
Educational attainment ^4,5^		0.100		0.149
Elementary and lower	1.2		6.3	
High school	1.7		7.4	
College and higher	1.2		7.2	
Marital status		0.001		<0.001
Single	0.4		3.1	
Married	1.6		8.1	
Others	1.9		8.3	
Employment status		0.023		0.068
Employed	1.1		6.6	
Unemployed	1.7		7.6	
Household size		0.327		0.052
1–3	1.6		7.8	
4–6	1.3		6.7	
≥7	1.2		6.3	
Wealth quintile		0.015		0.001
Poorest	0.7		4.7	
Poor	1.2		6.6	
Middle	1.3		7.1	
Rich	1.8		7.6	
Richest	1.8		8.4	
Current smoker ^4,5^		0.007		0.001
Yes	0.7		4.1	
No	1.6		8.1	
Current alcohol drinker ^4,5^		0.006		0.009
Yes	0.9		6.1	
No	1.9		8.0	
Physical activity ^4,5^		0.140		0.629
Low	1.6		6.9	
High	1.3		7.1	

^1^ Data are weighted percentage (%). ^2^ Ow/Ob + Ane: overweight/obesity and anemia. ^3^ Ow/Ob + Ane/VAD/Iod-Ins: overweight/obesity and anemia or vitamin A deficiency or iodine insufficiency. ^4^ Missing data in definition #1: educational attainment (*n* = 2), smoking and alcohol drinking status (*n* = 12), and physical activity (*n* = 13). ^5^ Missing data in definition #3: educational attainment (*n* = 4), smoking and alcohol drinking status (*n* = 64), and physical activity (*n* = 75).

**Table 4:** Logistic regression of factors associated with DBM in adults in the Philippines by two definitions ^1^.

Variables	Definition #1 ^2^			Definition #3 ^3^		
	(*n* = 237)			(*n* = 1241)		
	OR	95% CI	*p*-Value	OR	95% CI	*p*-Value
Female (ref = male)	1.77	1.18–2.67	0.041	1.47	1.23–1.75	0.008
Age group (ref = 20–39)						
40–59	1.84	1.28–2.67	0.022	2.11	1.77–2.51	0.000
≥60	1.34	0.84–2.14	0.280	1.70	1.34–2.17	0.007
Educational attainment						
Elementary and lower (ref)						
High school	1.38	0.97–1.96	0.135	1.33	1.11–1.58	0.026
College and higher	0.94	0.60–1.46	0.782	1.31	1.06–1.63	0.058
Marital status (ref = single)						
Married	2.64	1.44–4.85	0.026	2.10	1.65–2.67	0.002
Others	2.59	1.30–5.17	0.043	1.95	1.46–2.60	0.006
Unemployed (ref = employed)	0.81	0.58–1.13	0.268	0.95	0.81–1.10	0.496
Household size (ref = 1–3)						
4–6	0.85	0.62–1.16	0.347	0.81	0.69–0.94	0.043
≥7	0.80	0.54–1.20	0.332	0.87	0.72–1.06	0.223
Wealth quintile (ref = poorest)						
Poor	1.57	0.90–2.72	0.172	1.45	1.13–1.86	0.031
Middle	1.64	0.93–2.90	0.149	1.50	1.16–1.95	0.029
Rich	2.52	1.52–4.17	0.016	1.63	1.25–2.13	0.015
Richest	2.67	1.54–4.62	0.017	1.81	1.36–2.40	0.009
Current smoker (ref = yes)						
No	1.36	0.85–2.18	0.259	1.62	1.28–2.05	0.010
Current alcohol drinker (ref = yes)						
No	1.33	0.94–1.88	0.165	0.93	0.80–1.08	0.368
Physical activity (ref = low)						
High	0.93	0.70–1.24	0.655	1.14	0.99–1.32	0.126

^1^ Values are odds ratios (ORs) and 95% confidence intervals (CIs). Adjusted models are binary logistic regression models that control for all covariates shown. ^2^ Definition #1: overweight/obesity and anemia. ^3^ Definition #3: overweight/obesity and anemia or vitamin A deficiency or iodine insufficiency.
